# A Mental Health Survey of Medical Professionals Working During the COVID-19 Pandemic at Shaukat Khanum Cancer Hospital, Lahore

**DOI:** 10.7759/cureus.28869

**Published:** 2022-09-06

**Authors:** Muhammad H Chatha, Sanaa A Khan, Wajahat Nazir Ahmed, Ahsun Khan, Muhammad Abu Bakar, Muhammad Asif

**Affiliations:** 1 Anesthesia and Pain Management, Shaukat Khanum Memorial Cancer Hospital and Research Center, Lahore, PAK; 2 Anesthesia and Critical Care, Shaukat Khanum Memorial Cancer Hospital and Research Center, Lahore, PAK; 3 Biostatistics and Epidemiology, Shaukat Khanum Memorial Cancer Hospital and Research Center, Lahore, PAK; 4 Psychiatry, Shaukat Khanum Memorial Cancer Hospital and Research Center, Lahore, PAK

**Keywords:** depression, healthcare provider, mood and anxiety, sleep problems, covid-19 outbreak

## Abstract

Introduction

The last months of 2019 saw the emergence of a novel coronavirus, SARS-COV-2, capable of causing widespread disease in humans. The rapid spread of this new disease culminated in one of the biggest pandemics in known history. The far-reaching social, economical, and health effects of this pandemic are still unfolding on a global scale.

Given the interconnectedness of social, environmental, and biological factors in manifesting psychiatric illnesses, it is fair to assume that the profound effects of this pandemic would likely increase the strain on mental healthcare systems.

The objective of this study was to assess the mental health burden amongst healthcare workers at Shaukat Khanum Memorial Cancer Hospital and Research Center (SKMCH & RC) at the start of the COVID-19 pandemic and to identify any differences in the mental health scores of anxiety, depression, and sleep disturbance for professionals directly involved in the care of COVID-19 patients as compared to those who were not.

Material and methods

This was an observational cross-sectional clinical study that used self-reported questionnaires after approval from the hospital's ethical board. The sample size was calculated based on a study published previously by Huang using a 23.04% incidence of anxiety in medical staff. Depression was quantified using the Patient Health Questionnaire-9 score (PHQ-9), anxiety by the Beck Anxiety Inventory, and sleep quality using the Pittsburgh Sleep Quality Index checklist (PSQI). A total of 221 healthcare workers who completed the questionnaires were included in the study and the results were analyzed using SPSS Statistics v. 23 (IBM Corp., Armonk, NY). Levene’s test was used to assess the equality of variances, and an independent sample t-test and chi-square test were applied for the comparison of means. A one-way ANOVA test was used to compare means across more than two groups.

Results

Of the 221 healthcare workers recruited in the study, 57% were males, and 43% were females. Among the sample, 43% of participants were doctors, 27.1% were nurses, and others were technicians and medical assistants.

It was observed that 50% of males and 36% of female healthcare workers experienced moderate to severe depression at the onset of the pandemic. Furthermore, 35% of males and 25% of females suffered from moderate to severe anxiety, and more than 80% of our study population reported poor quality of sleep.

Conclusion

The present study reported a high prevalence of anxiety levels, depressive symptoms, and poor sleep quality among the healthcare professionals working in SKMCH & RC Lahore during the COVID-19 pandemic irrespective of direct contact with COVID-19 patients in a healthcare setting.

## Introduction

At the beginning of December 2019, there was a sudden outbreak of the novel coronavirus in the city of Wuhan, Hubei province, China. Before any concrete steps could be taken, the virus spread rapidly across the world. The pandemic has evolved into one of the deadliest pandemics to afflict humanity, with over 64 million deaths worldwide accounting for a loss of 0.5% of the world population. There are 1,551,251 confirmed cases in Pakistan alone, with 30,470 deaths reported so far to the date of writing this paper [1]. These numbers are expected to rise by the time this article is published.

While the quick development and deployment of vaccination programs have helped contain the fallout of the pandemic to some extent, the challenge is far from over. With new mutations of the COVID-19 virus such as the Delta and Omicron variants and lack of vaccination coverage owing to both limitations in capacity as well as anti-vaccination societal attitudes, it is difficult to accurately chart a course for the future.

Due to the apparent health emergency, global economic recession, social distancing, lack of recreational activities, and uncertainty about the future, it is reasonable to anticipate a worsening of a historically neglected mental health crisis. Finding themselves in unfavorable, new social settings, it is unsurprising that people have struggled to adapt.

COVID-19 is known to cause both physical and mental health damage to health care professionals [2]. Healthcare workers, including doctors, nurses, and paramedical staff, are working on the frontlines against the pandemic. As is often the case, medical personnel dealing with such large-scale outbreaks are prone to psychological stress and mood changes, affecting their health [3]. Medical professionals are likely to be adversely affected by the fear of being a source of transmission to their immediate family and friends [4]. The fear of contagion may add to their stress.

Since the start of the outbreak, there have been numerous efforts internationally to see the psychological impact of the pandemic on healthcare workers, but we have not seen many such studies in Pakistan. In a study conducted in China, the incidence of anxiety in tertiary care centers among medical staff involved in treating COVID-19 patients is as high as 23.04%, with female staff being more vulnerable [5]. Another study involving multiple hospitals in China concluded that 44.6% of the staff experienced severe anxiety symptoms [6].

Healthcare workers, may in general, be at a higher risk of mental health-related disorders [7] and, according to a study conducted in China, be at a greater risk of having poor sleep quality owing to the stressful nature of their job [2]. As expected, sleep quality for medical staff treating COVID-19 was also more negatively affected than the general population [8].

The quality of sleep is a crucial indicator of good health [9]. It boosts the immune system to prevent infections and improves physical and mental health, which positively influences the performance of health care workers [8]. Some studies have established an association between both anxiety and depression with poor sleep quality [10]. In a different study, this correlation between anxiety, depression, and sleep quality was corroborated [11].

Caregivers managing COVID-19 patients are also at a greater risk of developing psychological distress and other unfavorable mental health symptoms [5]. This may lead to decreased efficiency in dealing with the situation at hand and may also cause long-lasting mental ailments, as seen in the SARS pandemic, where healthcare workers were seen to develop PTSD [3].

The debate regarding the impact of the COVID-19 pandemic on mental health diseases revealed gaping deficiencies in existing data regarding the prevalence of anxiety, depression, and sleep disturbance among health care professionals in Pakistan. Hence, there was a dire need to determine the mental health burden and address it accordingly. 

The objective of this study was to assess the mental health burden amongst health care workers at Shaukat Khanum Memorial Cancer Hospital and Research Center (SKMCH & RC), a tertiary healthcare center in the second-largest city of Pakistan, at the start of the COVID-19 pandemic and to identify any difference in the mental health scores of anxiety, depression and sleep disturbance for professionals directly involved in the care of COVID-19 patients as compared to those who were not.

## Materials and methods

This was an observational cross-sectional clinical study, conducted between April to July 2020 at SKMCH & RC, Lahore. Self-reported questionnaires were used to collect data after approval from the institutional review board. The sample size was calculated based on a study published previously by Huang (2020) using a 23.04% incidence of anxiety in medical staff [2]. A total of 221 healthcare workers who completed the questionnaires were included in the study.

Healthcare professionals working in the ICU, operating theaters, COVID inpatient ward, outpatient COVID screening units, emergency room, and the radiology department were recruited. Healthcare workers were asked if they were directly involved in the care of COVID-19 patients or not.

The process and purpose of the study were explained in person to the participants and informed written consent was taken in accordance with the hospital policies of the institutional review board. Data was collected from SKMCH & RC Lahore through printed survey forms distributed in person to health care workers. Details included in proforma comprised demographic data, Patient Health Questionnaire-9 score (PHQ-9), Beck Anxiety Inventory (BAI), and Pittsburgh Sleep Quality Index (PSQI). After completion, the forms were kept in a designated folder and the confidentiality of participants was maintained. Calculation of scores from the questionnaires was done and entered electronically in an Excel sheet. At the end of data collection, statistical analysis was done with help from the statistical department at SKMCH & RC.

Demographical variables recorded were age, gender, profession, marital status, education level, and monthly salary. We used the patient health questionnaire-9 form to screen for symptoms of depression among the study participants. This is a reliable questionnaire to assess patients for screening for depression [12-13]. The BAI was employed to assess the anxiety levels of the study participants. This inventory questionnaire is an established tool for the assessment of anxiety symptoms [14]. The quality of sleep among the participants was measured using the PSQI form, which is a reliable and valid indicator of sleep quality [15-16]. All these assessment questionnaires have been used and validated in the Pakistani population [17-21].

Data were analyzed using SPSS Statistics v. 23 (IBM Corp., Armonk, NY). Levene’s test was used to assess the equality of variances, an independent sample t-test and chi-square test were applied for comparison of means, and a one-way ANOVA test was used to compare means across more than two groups.

## Results

The characteristics of the participants are shown in Table 1. Of the 221 samples analyzed, 126 (57%) were males, 95 (43%) were females, and the mean (standard deviation) age of the participants was 29.21±6.053 years. Among the sample, 95 (43%) participants were doctors, 60 (27.1%) were nurses, and others were technicians and medical assistants. Around 52.9% of participants were married, and 47.1% of participants earned a monthly salary of 50,000-100,000 Pakistani rupees (PKR). The majority of the participants (78.7%) had a post-graduate degree, and 80.5% had direct contact with COVID-19 patients.

**Table 1:** Demographic characteristics PKR: Pakistani rupee

Demographic characteristics		
	Variables	Count (%)
Total surveyed		221 (100)
Gender	Male	126 (57)
	Female	95 (43)
Profession	Doctor	95 (43)
	Assistant	11 (5)
	Nurse	60 (27.1)
	Technician	55 (24.9)
Marital status	Divorced	1 (0.5)
	Married	117 (52.9)
	Single	103 (46.6)
Direct contact	No	43 (19.5)
	Yes	178 (80.5)
Salary bracket	Less than 50,000 PKR	76 (34.4)
	50,000-100,000 PKR	104 (47.1)
	100,000-200,000 PKR	26 (11.8)
	More than 200,000 PKR	15 (6.8)
Education	Up to intermediate levels	14 (6.3)
	Under-graduate	33 (14.9)
	Post-graduate	174 (78.7)

The scores of PHQ-9, BAI, and PSQI were stratified by gender, profession, salary, and contact with COVID-19 patients. The results are given in Table 2 and demonstrated in Tables 1-6. The mean scores of PHQ-9, BAI, and PSQI were 8.98, 16.43, and 7.99, respectively. On average, males had a higher score on PHQ-9 and the BAI scale. Compared with the healthcare professionals who did not have exposure to COVID-19 patients, the scores were elevated for those who had direct contact. Further, the participants with a monthly income of 50,000-100,000 PKR reportedly had high scores for PHQ-9 and BAI as compared to their other counterparts.

**Table 2:** PHQ-9, BAI, and PSQI mean scores during the COVID-19 outbreak in Pakistan PHQ-9: Patient Health Questionnaire-9; BAI: Beck Anxiety Inventory; PSQI: Pittsburgh Sleep Quality Index; PKR: Pakistani rupee

PHQ-9, BAI, and PSQI mean scores during the COVID-19 outbreak in Pakistan			
Variables	PHQ-9	BAI	PSQI
Overall mean	8.98	16.43	7.99
Stratified by gender (independent t-test)			
Male	9.42	17.31	7.98
Female	8.41	15.25	8.01
Stratified by profession (one-way analysis of variance)			
Doctor	8.23 ± 6.9	14.93 ± 16.5	8.07 ± 3.6
Assistant	11.55 ± 4.9	21.55 ± 14.2	8.27 ± 3.7
Nurse	9.97 ± 7.3	18.88 ± 15.6	8.25 ± 3.7
Technician	8.53 ± 6.6	15.31 ± 13.7	7.51 ± 3.7
Stratified by direct contact with COVID-19 Patients (Independent t-test)			
No	8.81	15.88	7.28
Yes	9.02	16.56	8.16
Stratified by monthly salary bracket (one-way analysis of variance)			
<50,000 PKR	8.32 ± 6.8	15.61 ± 14.3	7.42 ± 3.9
50,000-100,000 PKR	9.98 ± 6.4	17.59 ± 15.3	8.59 ± 3.4
100,000-200,000 PKR	8.35 ± 7.1	18.42 ± 19.3	8.35 ± 3.9
>200,000 PKR	6.47 ± 8.4	9.07 ± 13.4	6.20 ± 2.7

The prevalence of anxiety and depressive symptoms and the assessment of sleep quality were stratified by gender, occupation, and direct contact with COVID-19 patients. Salary findings are shown in Tables 3-5, respectively. The overall prevalence of high, moderate, and low anxiety levels was found to be 18.55%, 11.76%, and 69.68%. There was no statistically significant difference in the prevalence of anxiety levels by stratifying the variables under study. 

**Table 3:** Prevalence of anxiety levels in healthcare professionals of Pakistan during the COVID-19 outbreak PKR: Pakistani rupee

Prevalence of anxiety levels in healthcare professionals of Pakistan during the COVID-19 outbreak			
Variables	High Anxiety	Low Anxiety	Moderate Anxiety
Gender (Sig. 2-sided: 0.354) (chi-square test)			
Male	20.63%	65.87%	13.49%
Female	15.79%	74.74%	9.47%
Profession (Sig. 2-sided: 0.28) (chi-square test)			
Doctor	21.05%	71.58%	7.37%
Assistant	18.18%	54.55%	27.27%
Nurse	21.67%	65.00%	13.33%
Technician	10.91%	74.55%	14.55%
Direct contact with COVID-19 patients (Sig. 2-sided: 0.645) (chi-square test)			
No	23.26%	67.44%	9.30%
Yes	17.42%	70.22%	12.36%
Salary brackets in PKR (Sig. 2-sided: 0.547) (chi-square test)			
<50,000	14.47%	71.05%	14.47%
50,000-100,000	21.15%	68.27%	10.58%
100,000-200,000	26.92%	61.54%	11.54%
>200,000	6.67%	86.67%	6.67%
Total	18.55%	69.68%	11.76%

Further, as depicted in Table 4, the prevalence of moderately severe or severe depressive symptoms was found among 27.6% of the healthcare professionals. There was a statistically significant difference in the prevalence of depressive symptoms within the gender variable. Except for the moderately severe level, depressive symptoms were found to be higher in males. Compared with other salary ranges, severe depressive symptoms were significantly higher in participants lying in the salary bracket of 100,000-200,000, while moderately severe symptoms were higher for those earning a salary range of 50,000-100,000.

**Table 4:** Prevalence of depressive symptoms in healthcare professionals of Pakistan during the COVID-19 outbreak PKR: Pakistani rupee

Prevalence of depressive symptoms in healthcare professionals of Pakistan during the COVID-19 outbreak					
Variables	Mild	Minimal	Moderate	Moderately Severe	Severe
Gender (Sig. 2-sided: 0.016) (chi-square test)					
Male	18.25%	30.95%	23.81%	20.63%	6.35%
Female	29.47%	34.74%	7.37%	22.11%	6.32%
Profession (Sig. 2-sided: 0.248) (chi-square test)					
Doctor	28.42%	34.74%	9.47%	22.11%	5.26%
Assistant	18.18%	9.09%	36.36%	36.36%	0.00%
Nurse	20.00%	28.33%	21.67%	21.67%	8.33%
Technician	18.18%	38.18%	20.00%	16.36%	7.27%
Direct contact with COVID-19 patients (Sig. 2-sided: 0.508) (chi-square test)					
No	16.28%	37.21%	20.93%	16.28%	9.30%
Yes	24.72%	31.46%	15.73%	22.47%	5.62%
Salary brackets in PKR (Sig. 2-sided: 0.034) (chi-square test)					
<50,000	21.05%	38.16%	19.74%	14.47%	6.58%
50,000-100,000	28.85%	21.15%	18.27%	26.92%	4.81%
100,000-200,000	15.38%	46.15%	3.85%	23.08%	11.54%
>200,000	6.67%	60.00%	13.33%	13.33%	6.67%
Total	23.08%	32.58%	16.74%	21.27%	6.33%

Next, Table 5 demonstrates that 81.90% of the surveyed healthcare professionals had a poor quality of sleep with a PSQI score of greater than 5. While there were no statistically significant differences within the demographic variables, the findings indicate a slightly significant difference in the quality of sleep between different salary brackets. One-third of those earning above 200,000 PKR enjoyed a good quality of sleep which is relatively higher than those in lower salary brackets. On the other hand, the highest percentage of the participants who had a poor quality of sleep was found among the ones with a salary bracket of 100,000-200,000 PKR.

**Table 5:** Assessment of sleep quality in healthcare professionals of Pakistan during the COVID-19 outbreak PKR: Pakistani rupee

Table 5. Assessment of sleep quality in healthcare professionals of Pakistan during the COVID-19 outbreak		
Variables	Good sleep quality	Poor sleep quality
Gender (Sig. 2-sided: 0.945) (chi-square test)		
Male	18.25%	81.75%
Female	17.89%	82.11%
Profession (Sig. 2-sided: 0.733) (chi-square test)		
Doctor	20.00%	80.00%
Assistants	18.18%	81.82%
Nurse	13.33%	86.67%
Technician	20.00%	80.00%
Direct contact with COVID-19 patients (Sig. 2-sided: 0.328) (chi-square test)		
No	23.26%	76.74%
Yes	16.85%	83.15%
Salary brackets in PKR (Sig. 2-sided: 0.099) (chi-square test)		
<50,000	23.68%	76.32%
50,000-100,000	13.46%	86.54%
100,000-200,000	11.54%	88.46%
>200,000	33.33%	66.67%
Total	18.10%	81.90%

## Discussion

The percentage of healthcare workers with moderate to severe anxiety was 30.3% without any association with factors such as gender, profession, salary, or direct contact with COVID-19 patients in healthcare settings. Studies conducted on healthcare workers from Pakistan [22-23] have consistently shown a higher percentage of study subjects suffering from anxiety when compared to an estimated prevalence of 23% reported in a systematic review of 12 studies conducted on more than 33 thousand healthcare workers from other Asian countries [24]. It is to be noted, however, that the majority of data for this systematic review was collected from studies conducted in China and Singapore, countries with a significantly higher GDP than Pakistan, therefore economic factors cannot be excluded when it comes to explaining a 30% increase in symptoms of moderate to severe anxiety amongst healthcare workers from Pakistan.

A study from our neighboring country, India conducted on over 1000 healthcare workers concluded that 37% of healthcare workers were suffering from anxiety [25]. This is comparable to our results. It is to be noted that while our data were collected at the onset of the pandemic, this study was conducted from March to May 2020 when India was experiencing a rapid increase in COVID-19 cases culminating in a public health crisis worthy of making international headlines. It can be hypothesized that anticipation of a similar disaster would also have had an effect on anxiety levels amongst Pakistani healthcare workers, given that both countries share comparable population demographics, healthcare infrastructure, and sociopolitical dynamics. It can be hypothesized that as the pandemic evolved, the proportion of healthcare workers with clinical anxiety may have increased more than the levels reported in our study due to these events unfolding on the global healthcare front. Therefore it seems appropriate to advocate for continuous research regarding anxiety levels amongst this high-risk population to better quantify the extent of the problem and assess the actual impact of the COVID-19 pandemic on mental health.

Around 27.6% of respondents were found to be suffering from moderately severe or severe depression. There was an association between gender and a monthly salary with the development of symptoms of depression. Although globally, the prevalence of depression is higher in females as compared to males [26], this is not the first time an association has been found between male healthcare workers and the presence of depressive symptoms. It is to note that direct contact with COVID-19 patients did not lead to a significant difference in the prevalence of depressive symptoms in healthcare professionals.

There is a stark contrast when comparing our results with the general global population, with the prevalence of moderate to severe depression reported at 4.5% [27]. Our results are comparable to prevalence rates of depression amongst the general Pakistani population [28]. Furthermore, healthcare workers working in other low-income countries such as Bangladesh (27-30%) and India (27%) have similar rates of depression [29-30]. 

Only one-fifth of healthcare professionals surveyed reportedly experienced good quality of sleep. Sleep disorders are not uncommon among healthcare workers, and growing reports range from 21-65.5% about the prevalence of sleep difficulties [31]. However, during the current COVID-19, they are at an even increased risk of mental health issues and sleep problems [32]. In our study, the reported prevalence of poor sleep quality was 81.9%, indicating an increase in sleep disturbances among healthcare workers during the pandemic. This conforms to a cross-sectional study conducted by Abdulah and Musa, which included physicians from different medical settings during the COVID-19 outbreak and confirmed a negative impact on participants' sleep during this time [33]. In another study, the prevalence of poor sleep quality during the pandemic was 78.8%, comparable to our results [31]. The finding that a higher monthly salary was associated with good sleep quality underscores the prevalence of economic stress caused during the pandemic.

There are, nevertheless, certain limitations to the study. It was conducted specifically with healthcare workers, so its results may not be generalizable to other professions. All the respondents were from Lahore, a metropolitan city. The results could reveal different anxiety levels, depressive symptoms, and sleep quality as compared to semi-urban areas where the healthcare facilities differ. Moreover, the design of the study was cross-sectional, so the results should be interpreted cautiously.

## Conclusions

The present study reported a high incidence of anxiety levels, depressive symptoms, and poor sleep quality among the healthcare professionals working in SKMCH & RC Lahore during the COVID-19 pandemic. There was no significant difference between mental health scores for professionals directly involved in the care of COVID-19 patients as compared to those who were not. Prospective studies using experimental or longitudinal designs with an increased sample size should be conducted to examine the long-term psychological impact of the COVID-19 outbreak among healthcare workers in Pakistan. Further, continuing investigation of psychological consequences with regards to outbreaks of such life-threatening epidemics should be conducted on a routine basis as part of preparedness efforts globally.

## Figures and Tables

**Figure 1:**
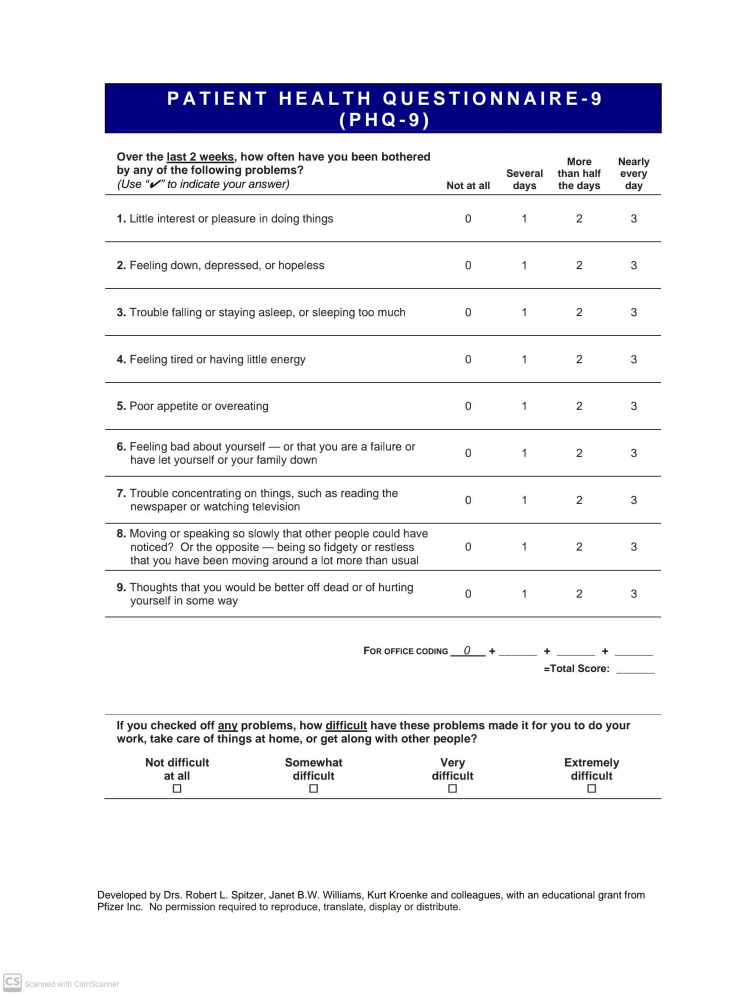
Patient Health Questionnaire-9

**Figure 2:**
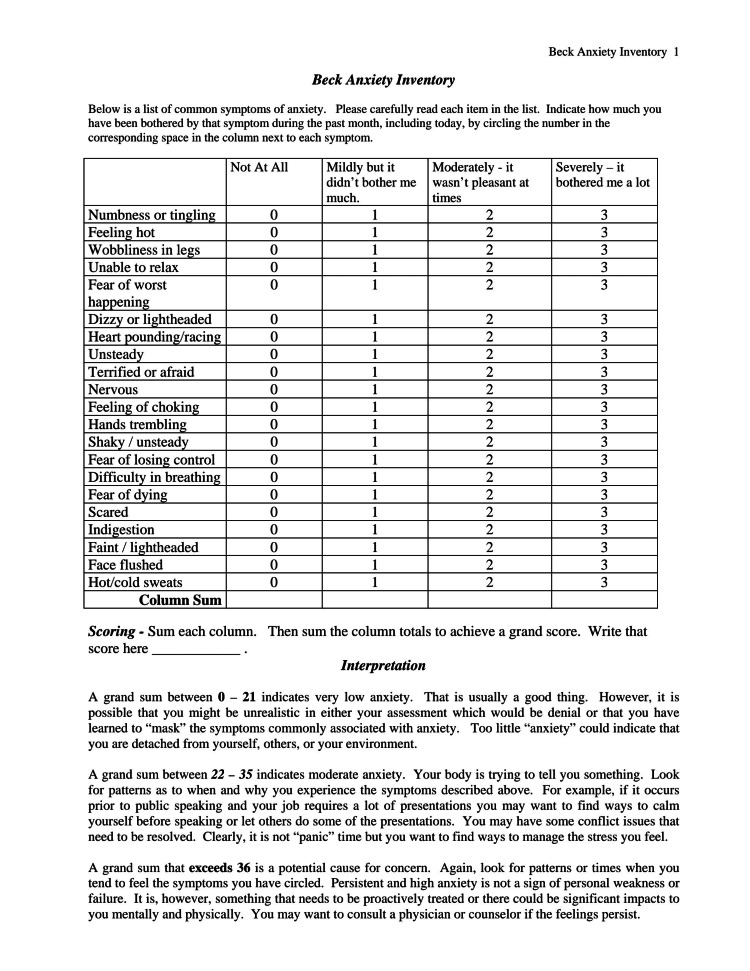
Beck Anxiety Inventory

**Figure 3:**
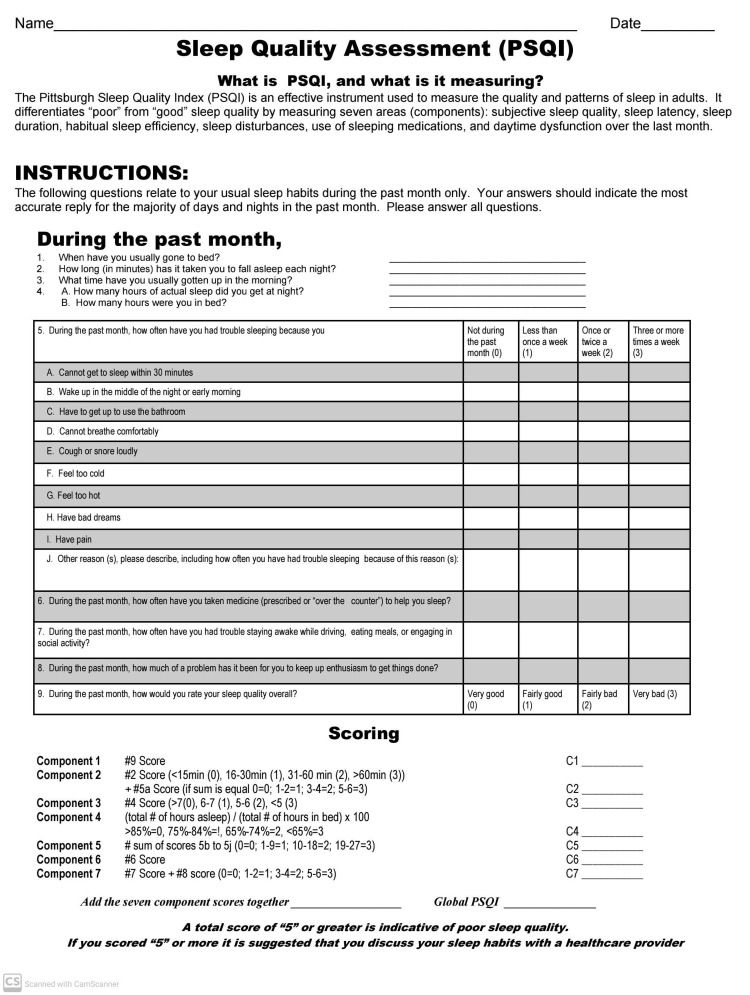
Pittsburgh Sleep Quality Index

## Appendices

Table 6 shows the demographic details included in the questionnaire; Figures 1-3 show the Patient Health Questionnaire-9 score (PHQ-9), the Beck Anxiety Inventory (BAI), and the Pittsburgh Sleep Quality Index checklist (PSQI). 

**Table 6:** Demographic details

Age	
Gender	
Profession	
Marital status	
Direct contact to COVID-19	
Diagnosed pre-existing mental health condition	
Exempt from attending duties in hospital	
Education level (select one)	High school, college, undergraduate, Post-graduate
Salary per month	< 50,000 50,000-100,000 100,000-200,000 200,000-300,000 300,000-400,000 400,000-500,000 > 500,000
